# Planning and preparing for public health threats at airports

**DOI:** 10.1186/s12992-018-0323-3

**Published:** 2018-03-07

**Authors:** Greg Martin, Mairin Boland

**Affiliations:** 1 Health Services Executive, 25-27 Middle Gardiner Street, Dublin 1, DO1 A4A3 Ireland; 2 Department of Public Health, Health Services Executive East, Dublin 8, Ireland

**Keywords:** Port health, Infectious disease, Travel, Globalization, Pandemic, Airport, Preparedness, Exercise, Health protection

## Abstract

The ever-increasing speed and scope of human mobility by international air travel has led to a global transport network for infectious diseases with the potential to introduce pathogens into non-endemic areas, and to facilitate rapid spread of novel or mutated zoonotic agents.

Robust national emergency preparedness is vital to mitigate the transmission of infectious diseases agents domestically and to prevent onward spread to other countries. Given the complex range of stakeholders who respond to an infectious disease threat being transmitted through air travel, it is important that protocols be tested and practised extensively in advance of a real emergency. Simulation exercises include the identification of possible scenarios based on the probability of hazards and the vulnerability of populations as a basis for planning, and provide a useful measure of preparedness efforts and capabilities.

In October 2016, a live simulation exercise was conducted at a major airport in Ireland incorporating a public health threat for the first time, with the notification of a possible case of MERS-CoV aboard an aircraft plus an undercarriage fire. Strengths of the response to the communicable disease threat included appropriate public health risk assessment, case management, passenger information gathering, notification to relevant parties, and communication to passengers and multiple agencies.

Lessons learned include:

o Exercise planning should not be overly ambitious. In testing too many facets of emergency response, the public health response could be deprioritised.

o The practical implementation of communication protocols in a real-time exercise of this scope proved challenging. These protocols should continue to be checked and tested by desk-top exercises to ensure that all staff concerned are familiar with them, especially in the context of staff turn-over.

o The roles and responsibilities of the various agencies must be clear to avoid role confusion.

o Equipment and infrastructure capacities must be considered and in place in advance of an actual incident or test, for example whether or not cell phone signals require boosting during a major event.

Importantly, exercises bring together individuals representing organisations with different roles and perspectives allowing identification of capabilities and limitations, and problem solving about how to address the gaps and overlaps in a low-threat collaborative setting.

The Public Health threat from a novel zoonotic outbreak with pandemic potential being propagated though air travel is more than an abstract notion, but rather an issue of serious concern that deserves our utmost attention. The emergence of severe acute respiratory syndrome (SARS) in 2003 demonstrated the potential for a disease to suddenly appear, spread and threaten the health, economic and social life of people all over the world [1]. The frequency with which new pathogens are emerging is increasing with time [2].Within living memory we have seen the emergency of HIV, Ebola Virus Disease, SARS, H1N1 influenza, MERS-CoV and other novel zoonotic infections affecting people and crossing geopolitical boundaries of nation states. Most pandemics are caused by viruses that originate in animals and infect humans due to ecological, behavioural and socioeconomic changes. Over 60% of the roughly 400 emerging infectious diseases that have been identified since 1940 are zoonotic [2].

The ever-increasing speed and scope of human mobility by international air travel has led to a global transport network for infectious diseases [3] with the potential to introduce pathogens into non-endemic areas [4–6], and to facilitate rapid spread of novel or mutated zoonotic agents such as the H1N1 pandemic virus in 2009 [5, 7–9]. Other pathogens that have been documented to have been spread through air travel include TB, meningococcal disease, measles, salmonellosis, shigellosis, cholera, viral enteritis, malaria, dengue, yellow fever and smallpox (before eradication) [10].

Adding to the risk associated with the emergence of zoonotic outbreaks, is a growing concern that non-state actors will deploy a biological weapon in an act of terrorism. The US Commission on the Prevention of Weapons of Mass Destruction Proliferation and Terrorism in 2008 stated that it is more likely than not that a biological weapon will be used by a non-state actor in the foreseeable future [11]. Since the publication of that report we have seen the advent of CRISPR-Cas9, an affordable ready-to-use gene editing technology [12]. It is thought that genome editing could potentially be used to deliberately create an airborne pathogen to be used as a biological weapon. This threat of airborne pathogens designed with short latency periods and long incubation periods to ensure global transmission through air travel networks represents an important risk.

Under International Health Regulations (IHR) 2005 [13] 196 countries worldwide have committed to strengthen response to serious cross borders health threats. At designated points of entry (airports, sea ports and ground crossings) signatory states maintain systems and infrastructure to prevent, monitor, detect, report and respond to public health threats. EU Decision 1082 on cross border threats [14] brings European legislation in line with IHR, and encompasses preparedness and response planning.

In Ireland, Infectious Disease Shipping and Aircraft Regulations were brought into force in 2008 and 2009, respectively. In 2009, following recommendations from an IHR Assessment Group Report, a Medical Officer of Health (MOH) Port Health Committee was established. This committee acts as a forum for sharing experience and knowledge regarding preparedness for, and response to, communicable disease incidents at points of entry, and organises training and desktop exercises to test the guidelines that have been produced. In addition, the committee works in tandem with the national multidisciplinary Health Services Executive Port Health network to progress interoperability and cross sectoral working.

Given the complexity and the range of stakeholders involved with responding to an infectious disease threat being transmitted through air travel, it is important that protocols be tested and practised extensively in advance of a real emergency. We focus here on capability to respond to a public health threat, namely, the actions the public health system is capable of taking to effectively identify, characterise and respond to an emergency. The capacities, i.e. the resources in terms of infrastructure, policies and procedures and skilled personnel are also a vital component [15]. Exercises test capabilities and improve response [16], and should be a regular element of all national emergency preparedness. In recent years, great strides have been made in terms of emergency preparedness through assessment of potential hazards that public health systems may face [17], refining strategies for resource allocation [18], integrating preparedness training into practice [19] and sharing of lessons learned from After Actions Reports [20].

Exercises include the identification of possible scenarios based on the probability of hazards and the vulnerability of populations as a basis for planning [21] and have been shown to be a useful tool to measure preparedness efforts [22, 23]. The absence of actual testing is likely to negate even the best of abstract plans [21, 24]. Conducting exercises provides a valuable forum to judge the adequacy of legal authorities, policies and procedures for dealing with emergencies at the state and local levels [16, 25]. Simulation exercises are a form of practice, training or evaluation of capabilities involving the description (simulation) of an emergency. Simulations enable responders to practise their roles and functions and can help to develop, assess and test functional capabilities of systems, procedures and mechanisms to respond to public health emergencies, identifying strengths and gaps [26].

A large-scale simulated emergency exercise is conducted by one of Ireland’s major international airports every three years to strengthen interagency emergency preparedness, and in October 2016 included a Public Health threat for the first time, with the MOH port health committee involved in planning. The live real-time exercise included the notification of a possible case of MERS-CoV aboard an aircraft carrying 81 people. The exercise also included an undercarriage fire and a subsequent emergency landing of the aircraft. This field exercise was designed to test the public health response in the context of a complex emergency involving multiple response agencies. The exercise involved over 200 participants at the airport including airport and airline staff and management, fire officers, police, health services responders including national ambulance service, media, regional Public Health and other relevant sites like the National Isolation Unit for infectious diseases (NIU) and Health Protection Surveillance Centre (HPSC), observers, evaluators and passenger volunteers. Careful planning took place over six months, with development of materials, scenario writing, and interrogation of response protocols, leading to the set-up of three different field sites: the operation control centre, the passenger reception area and the plane and runway. Much of the substance of the exercise and the approach reflected the WHO Simulation Guide approach [26]. Immediately following the exercise, a ‘hot’ debrief took place to clarify the main priorities and other smaller issues, to enable immediate influence on future response and to reduce recall challenges.

Emergency exercises are only valuable to the extent that we learn from them and implement our findings into day-to-day practice. The implications of this exercise for public health preparedness can be considered in the context of the Donabedian model [27] which contends that information about structure, process and outcomes can be collected to make inference about the quality of a service.

## Structure

Structural issues that contribute toward emergency preparedness include the physical environment, available infrastructure, equipment, documentation and information. Readiness was demonstrated with detailed airport public health alert protocols, identification of a landing area and passenger reception area, presence of documentation and equipment on site for rapid assessment, and information leaflets for passengers and crew. Structural gaps highlighted were restricted access to a fax machine, high levels of background noise in the passenger reception area (making telephone conversations difficult), a lack of appropriate space to conduct an interview with contacts and potential issues with the strength of cell/ mobile phone signals.

## Process

Public Health processes were carried out efficiently and with a high level of fidelity to protocols following the arrival of passengers and crew at the passenger reception area. Process failures that delayed the activation of public health measures included some lack of adherence to communication protocols and the prioritisation of the non-public health aspects of the exercise by some other agencies.

## Outcomes

Outcomes of interest included:

- the fact that by the end of the exercise, all passengers were correctly assigned into risk stratified groups,
- all passengers were given information as appropriate,
- passenger locator forms were correctly filled by ‘at-risk’ passengers and collected by Public Health doctors,
- the Health Protection Surveillance Centre (Ireland’s National Focal Point under IHR) was notified of the incident,
- Environmental Health Services were informed of the incident,
- the National Isolation Unit was contacted and provided with appropriate information and a multiagency media communication was agreed.

The issues identified during this exercise illustrate the complex nature of a multiagency response to an incident. While the exercise highlighted important gaps in communication capacity between agencies, the positive outcomes that emerged demonstrated an overall effective response to an emergency by Public Health doctors. Outcomes and issues to prioritise to improve preparedness were fed back to all stakeholders in writing and at meetings, and are the subject of the next iteration of exercises comprising desk-top scenarios in 2018.

Lessons learned include:

- Exercise planning should not be overly ambitious. By trying to test too many facets of the emergency response protocols, the public health response can be deprioritised,
- The practical implementation of communication protocols in a real time exercise of this scope proved challenging. These protocols should continue to be checked and tested in desk-top exercises often to ensure that all staff concerned are familiar with them, especially in the context of staff turn-over,
- The roles and responsibilities of the various agencies involved needs to be clear. In the chaos of an incident it is easy for role confusion to set in,
- Equipment and infrastructure must be in place and must have been thought about before an actual incident. Whether or not cell phone signals are available on site or require boosting and how precisely documentation like the flight manifest are going to be communicated to Public Health officials have to be in place well in advance of an incident.

Emergency preparedness should be a multisector activity and take into account the vulnerabilities, infrastructure and capabilities of a country or region’s health systems [21]. Importantly, exercises bring together individuals representing organisations with different roles and perspectives [23] allowing them to identify each other’s capabilities and limitations, and problem-solve about how to address the gaps and overlaps in a low-threat collaborative setting [28]. The benefits of engaging in multisectoral exercises include a raised awareness of public health threats, identification of priorities and improved working relationships between agencies [23]. Post-exercise review meetings and reports allow for sharing of the subjective experience of the participants and agencies, and objective measures against predetermined exercise aims.

While improving the national response capabilities in Ireland is an essential component of health protection at ports, individual robust national protocols are not enough. Weak health systems in many low-income countries increase the likelihood of spreading outbreaks with the potential to cross borders and threaten global health security. Notwithstanding the humanitarian impetus to provide health systems strengthening support to poorer countries, high-income countries in their own interest should make every effort to ensure that health protection protocols are in place and exercised appropriately the world over. The European Commission Joint Action on Preparedness for Health Threats at points of entry [29] 2017 emphasises joint testing both within the EU and with neighbouring countries.

A poorly designed or executed exercise may do more harm than good if it leads to a false sense of security and results in poor performance during an actual emergency [30]. Planning, executing and reporting on exercises to improve emergency preparedness should be high on the agenda of public health departments in all countries.
